# APHRODITE: A Compact Lab-on-Chip Biosensor for the Real-Time Analysis of Salivary Biomarkers in Space Missions †

**DOI:** 10.3390/bios14020072

**Published:** 2024-01-30

**Authors:** Lorenzo Nardi, Nithin Maipan Davis, Serena Sansolini, Thiago Baratto de Albuquerque, Mohcine Laarraj, Domenico Caputo, Giampiero de Cesare, Seyedeh Rojin Shariati Pour, Martina Zangheri, Donato Calabria, Massimo Guardigli, Michele Balsamo, Elisa Carrubba, Fabrizio Carubia, Marco Ceccarelli, Michele Ghiozzi, Liyana Popova, Andrea Tenaglia, Marino Crisconio, Alessandro Donati, Augusto Nascetti, Mara Mirasoli

**Affiliations:** 1 School of Aerospace Engineering, Sapienza University of Rome, Via Salaria 851, I-00138 Rome, Italy; nithin.maipandavis@uniroma1.it (N.M.D.); sansolini.1873557@studenti.uniroma1.it (S.S.); baratto.thiago@gmail.com (T.B.d.A.); mohcine.laarraj@uniroma1.it (M.L.); augusto.nascetti@uniroma1.it (A.N.); 2 Department of Information Engineering, Electronics and Telecommunications, Sapienza University of Rome, Via Eudossiana 18, I-00184 Rome, Italy; domenico.caputo@uniroma1.it (D.C.); giampiero.decesare@uniroma1.it (G.d.C.); 3 Department of Chemistry “Giacomo Ciamician”, Alma Mater Studiorum—University of Bologna, Tecnopolo di Rimini, Via Dario Campana 71, I-47922 Rimini, Italy; seyedeh.shariatipou3@unibo.it (S.R.S.P.); martina.zangheri2@unibo.it (M.Z.); mara.mirasoli@unibo.it (M.M.); 4 Department of Chemistry “Giacomo Ciamician”, Alma Mater Studiorum—University of Bologna, Via Selmi 2, I-40126 Bologna, Italy; donato.calabria2@unibo.it (D.C.); massimo.guardigli@unibo.it (M.G.); 5 Interdepartmental Centre for Industrial Aerospace Research (CIRI AEROSPACE), Alma Mater Studiorum—University of Bologna, Via Baldassarre Canaccini 12, I-47121 Forlì, Italy; 6 Kayser Italy S.r.l. Unipersonale, Via di Popogna 501, I-57128 Livorno, Italy; m.balsamo@kayser.it (M.B.); e.carrubba@kayser.it (E.C.); f.carubia@kayser.it (F.C.); m.ceccarelli@kayser.it (M.C.); m.ghiozzi@kayser.it (M.G.); l.popova@kayser.it (L.P.); a.tenaglia@kayser.it (A.T.); a.donati@kayser.it (A.D.); 7 Agenzia Spaziale Italiana (ASI), Italian Space Agency, Via del Politecnico, I-00133 Rome, Italy; marino.crisconio@asi.it

**Keywords:** lab-on-chip, chemiluminescence, hydrogenated amorphous silicon photosensors, biosensor, International Space Station, immunoassay

## Abstract

One of the main challenges to be faced in deep space missions is to protect the health and ensure the maximum efficiency of the crew by preparing methods of prevention and in situ diagnosis. Indeed, the hostile environment causes important health problems, ranging from muscle atrophy, osteopenia, and immunological and metabolic alterations due to microgravity, to an increased risk of cancer caused by exposure to radiation. It is, therefore, necessary to provide new methods for the real-time measurement of biomarkers suitable for deepening our knowledge of the effects of space flight on the balance of the immune system and for allowing the monitoring of the astronaut’s health during long-term missions. APHRODITE will enable human space exploration because it fills this void that affects both missions in LEO and future missions to the Moon and Mars. Its scientific objectives are the design, production, testing, and in-orbit demonstration of a compact, reusable, and reconfigurable system for performing the real-time analysis of oral fluid samples in manned space missions. In the frame of this project, a crew member onboard the ISS will employ APHRODITE to measure the selected target analytes, cortisol, and dehydroepiandrosterone sulfate (DHEA-S), in oral fluid, in four (plus one additional desired session) separate experiment sessions. The paper addresses the design of the main subsystems of the analytical device and the preliminary results obtained during the first implementations of the device subsystems and testing measurements on Earth. In particular, the system design and the experiment data output of the lab-on-chip photosensors and of the front-end readout electronics are reported in detail along with preliminary chemical tests for the duplex competitive CL-immunoassay for the simultaneous detection of cortisol and DHEA-S. Different applications also on Earth are envisaged for the APHRODITE device, as it will be suitable for point-of-care testing applications (e.g., emergency medicine, bioterrorism, diagnostics in developing countries, etc.).

## 1. Introduction

APHRODITE is an analytical device jointly developed by the School of Aerospace Engineering (SIA) of Sapienza University of Rome, the Department of Chemistry “Giacomo Ciamician” of the University of Bologna, and Kayser Italia srl. It is funded by the Italian Space Agency (ASI), with the objective of producing a technological demonstrator to be launched to the International Space Station (ISS) for the determination of salivary biomarkers through an innovative biosensor. The core system is a lab-on-chip (LoC) with integrated thin-film sensors in hydrogenated amorphous silicon (a-Si:H) and is able to host dual-analytes competitive chemiluminescence (CL) immunoassay.

One of the main challenges to be faced in future manned deep space missions is to protect the health and ensure the maximum efficiency of the crew by preparing methods of prevention, in situ diagnosis, and/or through telemedicine and countermeasures aimed at contrasting the typical dysfunctions and pathologies of long-duration spaceflight [1]. Indeed, the hostile environment causes important health problems, ranging from muscle atrophy, immunological and metabolic alterations, and increased risk of cancer, caused by the combined effect of microgravity and the exposure to cosmic radiation [2,3,4,5].

The ISS is equipped with advanced medical kits that have evolved significantly from earlier space missions. These kits include over 190 different medications, covering a broad range of medical needs from minor ailments to more serious conditions. These kits contain a wide range of pharmaceuticals, including analgesics, decongestants, sleep aids, and medications for more serious conditions. The ISS medical system, guided by the National Aeronautics and Space Administration (NASA) Human Research Program’s Exploration Medical Capability, employs a Model-Based Systems Engineering approach, enhancing its readiness for deep space missions. Astronauts aboard the ISS also undergo regular health monitoring, and the medical procedures aboard the station are well-documented, allowing the crew to contact the flight surgeon at ground control for guidance in case of medical issues. The complexity of managing these medical kits is significant, given the logistical challenges of resupplying the ISS with essential medicines and equipment. As for the frequency of astronauts getting sick, it is not common for astronauts to fall seriously ill during space missions. The environment aboard the ISS is tightly controlled to minimize health risks, and astronauts undergo extensive health checks before their missions. However, minor health issues like muscle aches, headaches, or sleep disturbances can occur, given the unique stresses of living in a microgravity environment. The ISS’s advanced medical kits and health monitoring systems are designed to address such issues promptly and efficiently [6,7].

Current diagnostics on the International Space Station (ISS) represents a significant leap in space medicine, integrating advanced biosensors, ultrasound technology, and molecular diagnostics to monitor astronaut health during long-duration missions. Emphasizing portability and the adaptability to microgravity, these tools include wearable biosensors for real-time physiological monitoring, comprehensive ultrasound for diverse clinical assessments, and molecular diagnostics for detecting health risks and infectious agents. Noteworthy advancements include the first DNA sequencing in space, underscoring the ISS’s role as a cutting-edge medical laboratory. These developments are critical for ensuring the well-being of astronauts, highlighting the role of innovative diagnostic technologies in space exploration [8].

The alterations of the immune system, closely linked to the psychophysical stress typical of long-duration space missions, are potentially very dangerous, especially in the confined environment of a spacecraft [2]. Reduced immune response can lead to the reactivation of latent viral infections (e.g., herpes viruses) and expose astronauts to the risk of bacterial, fungal, viral, and parasitic infections. In fact, it is important to remember that where there are humans, microorganisms are also present, and it is impossible to guarantee the absolute absence of pathogens. A study conducted on the Space Shuttle showed that during a 12-day mission, the presence of bacteria in the spacecraft’s atmosphere increased by 300% [9]. Furthermore, microgravity can enhance the growth rate and production of secondary metabolites by microorganisms, as well as alter the susceptibility of opportunistic pathogens to antibiotics and their virulence [10,11,12,13].

In addition to infection control, the immune system is also responsible for eliminating non-functional or dysfunctional tissue cells (e.g., tumor cells). Failing to maintain adequate immunity can therefore increase the risk of cancer development. It is, therefore, necessary to provide new methods for the real-time measurement of suitable biomarkers in order to deepen our understanding of the effects of spaceflight on the immune system’s balance and to enable the health monitoring of astronauts during long-duration missions [14,15,16].

Several countermeasures are in practice or under consideration to prevent and counteract immune system dysregulation in long duration spaceflight, such as exercise, stress relieving strategies, dietary supplements, and pharmacological intervention [17]. It appears more and more evident that a personalized approach could provide the best results, tailoring interventions based on the pre-flight evaluation of each crewmember’s unique immunological susceptibilities and the in-flight monitoring of immune parameters [17]. In the present study, we employed saliva as an easily and non-invasively collected sample, which can be analysed without pre-analytical sample preparation [18]. In particular, we focused on two biomarkers, chosen among those that are indicators of stress and immune system dysregulation. To this end, saliva has been analysed in astronauts to evaluate the presence of latent viruses (such as Epstein–Barr virus, herpes simplex virus-1, and Varicella zoster virus) [19], stress biomarkers (such as cortisol, α-amylase, and DHEA) [14], salivary antimicrobial proteins (such as secretory IgA, LL-37, α-defensins, lactoferrin, α-amylase, and lysozyme) [14], and cytokines [20].

In the context of clinical chemistry analyses on crewmembers, it also has to be highlighted that, except for the few Apollo missions, all human activity in space has so far taken place in low orbit, so a return to Earth is always possible in circumstances where this is necessary. In the case of biomedical research, samples collected on the ISS are generally stored at a controlled temperature until they are sent to Earth for laboratory analysis [14,15,19,20,21,22]. This scenario will obviously not be viable for future manned missions beyond low earth orbit (LEO) [8]. The development of diagnostic tools that can be used by astronauts themselves during a space mission is therefore a relatively recent priority in aerospace research [23]. Analytical devices suitable for spaceflight must minimally utilize onboard resources and operate under microgravity conditions, which also complicates the collection and management of biological samples. Additionally, these devices must function in the presence of relatively high levels of radiation [24,25]. Moreover, while specific studies are yet to be reported, it is reasonable to assume that in microgravity, the distinct conditions of diffusion processes and the absence of convection may also modify interactions between nanoparticles. The dynamic behavior of nanoparticles, typically governed by gravitational forces and sedimentation, could be influenced, thereby impacting the stability of their suspensions [26].

A good design choice for such systems that need to overcome all the presented issues is the implementation of LoC technology, which permits the miniaturization of the device by exploiting microfluidic approaches. Some of its most important advantages are the improvement in the efficiency of the analytical process, regarding the sample size, response time, cost, analytical performance, process control, integration, analytical productivity, and automation. Such devices have already been used on Earth in numerous biomedical applications and studies [27,28,29,30]. Even if the development of miniaturized microfluidic analytical devices is a rapidly expanding research field, only a few instances exist where such devices have been employed in space missions. In 2006, NASA tested the LOCAD-PTS on board the ISS, which is a portable microbial monitoring device based on lateral flow technology [31]. In 2013, the Microflow device, a fiber-optic-based flow cytometry platform, was employed onboard the ISS in order to analyze cells and hormones in blood [32]. Between 2016 and 2017, the MinION, a lab-on-chip device for nucleic acid sequencing utilizing nanopores, was deployed on the ISS [33].

Recently, the IN SITU Bioanalysis project led to the development of an autonomous analytical device suitable for the measurement of the salivary concentration of cortisol, a stress biomarker, directly on board the ISS [1,34]. The analytical system, designed to perform CL immunoassays, comprised a CL reader and a series of disposable cartridges containing all the necessary reagents for the analysis. This setup allowed astronauts to perform chemical-clinical analyses on biological samples for the first time, achieving detection limits on the order of ng/mL. However, this device is no longer competitive for future space missions due to its bulkiness, the significant storage space required for temperature-controlled reagents, and the substantial astronaut time needed for its operation.

An advance in this area was made through AstroBio CubeSat (ABCS) whose payload was a LoC based on CL measurements capable of autonomously conducting biological experiments; this was the first technological demonstrator that flew successfully in July 2022 [35].

Finally, within the framework of the PLEIADES project, an integrated analytical system based on LoC technology capable of performing bioassays with CL detection was developed. Designed for space applications, this system offered numerous advantages, such as compact size, reduced mass, the low consumption of reagents and samples, and low detection limits [28]. However, the main limitation of PLEIADES, as well as most microfluidic devices, pertains to the functionalization of channels with bio-specific molecules. Functionalized channels can typically be used only once unless the chip undergoes regeneration through lengthy and complex in-channel cleaning procedures and functionalization protocols [36]. The most commonly adopted solution is to replace the chip after each analysis, but the use of disposable devices results in an increase in payload mass and volume, which are quite a costly resource in space applications.

In this paper, which is an extended paper of the extended abstract presented at the SEIA 23 Conference [37], we report the system design and preliminary results of an innovative platform conceived to enhance space exploration by permitting the detection of numerous target analytes of interest in microgravity with a new design that permits its reusability and reconfigurability.

## 2. System Overview

**Figure 1:**
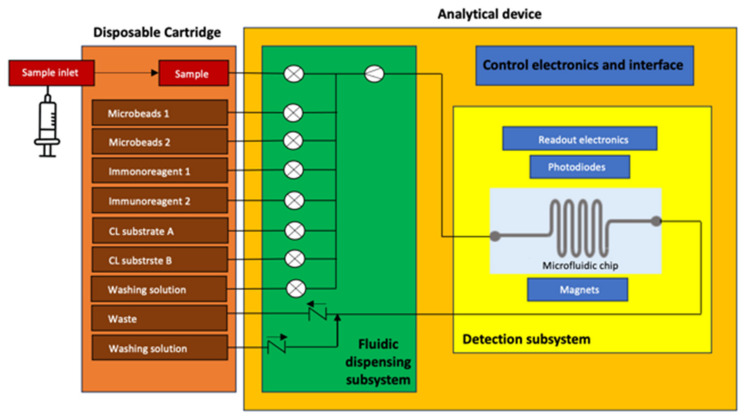
APHRODITE block diagram [37].

APHRODITE is a compact and versatile biosensor (Figure 1) to be launched on the ISS for carrying out the analyses of astronaut saliva for a quantitative determination of the levels of cortisol and dehydroepiandrosterone sulfate (DHEA-S), chosen as biomarkers for stress and alterations of the immune system. Cortisol has a key role in stress responses: it reflects hypothalamic–pituitary–adrenal (HPA) axis activity, and it can have immunosuppressive effects. DHEA and its sulfate (DHEA-S) also play a role in the stress system: they have anti-glucocorticoid properties, and they counterbalance the negative effects of cortisol. In particular, due to the opposing effects of these hormones, an elevated cortisol/DHEA(S) ratio is considered an indicator of immune system dysregulation [14].

The APHRODITE device format is properly designed to achieve the ultrasensitive quantification of the target biomarkers present in sub-molar quantities in a non-invasive way. The biosensor is integrated with easy-to-use and portable instrumentation with highly miniaturized accessories and microfluidics that permit automatic operation and reconfigurability to perform different analyses.

The key technical solution of APHRODITE is based on the combination of a microfluidic chip with the use of functionalized magnetic microbeads (MBs). Analytical methods based on specific recognition elements, like immunoassays, are suitable for the exploitation of LoC devices thanks to their high specificity and sensitivity, which make it possible to detect analytes even at low concentrations in a small volume of complex matrices. Immunoassays are well-established bioanalytical techniques, based on antibody–to–antigen binding, and used extensively in clinical chemistry to determine a wide range of analytes. In APHRODITE, analyte-specific capture antibodies are immobilized on MBs [38,39,40,41,42,43], which are then loaded in the chip channel, kept in position using permanent magnets during assay execution, and then eliminated by removing the magnetic field and washing, leaving the system clean and ready for the next analysis. Therefore, it is not necessary to functionalize the internal surface of the channels of the microfluidic chip like in most microanalytical devices, for example, in the PLEIADES project [28]. Finally, optical detection based on the measurements of CL photon emission by means of a-Si:H photodiodes has been chosen since it represents an ideal approach for LoC application because it enables the sensitive detection of the CL signal with a simple instrumental set-up [44,45] and it has already been validated in microgravity [35].

Currently, the IMMUNOLAB device is available onboard the ISS for immunoassays execution exploiting fluorescence microscopy detection [46]. One advantage of APHRODITE over IMMUNOLAB is related to the choice of the detection principle. Thanks to the use of enzyme-catalyzed CL detection instead of fluorescence, the APHRODITE device can take advantage of enzyme amplification and high signal-to-noise value to reach lower limits of detection (LOD). In addition, CL allows the design of simpler and smaller devices for signal detection with respect to fluorescence, which requires complex optical systems including excitation light sources and optical filters. In addition, the development of a LoC device with integrated thin-film photosensors provides compact size, minimal weight, high photon collection efficiency, intrinsic mechanical stability against vibrations, and limited data budget with respect to imaging detection [28]. On the other hand, the IMMUNOLAB has the advantage of enabling pre-analytical sample preparation. While sample preparation is not required for saliva analysis, future device developments will be required to implement sample preparation in APHRODITE in order to expand the range of detectable biomarkers to those contained in blood and urine.

### 2.1. Assay Principle

Cortisol and DHEA-S are quantified in the oral fluid sample using a CL-based immunoassay. This assay relies on biospecific reagents, specifically an antibody tailored to bind the target analyte and an enzyme conjugate wherein the target molecule is covalently linked to an enzyme that is capable of catalyzing a CL reaction (e.g., horseradish peroxidase, HRP).

A solid-phase competitive immunoassay format was employed, where the analyte in the sample (e.g., cortisol) competes with the enzyme conjugate (e.g., cortisol-HRP) for the binding to a limited quantity of the specific antibody (e.g., anti-cortisol IgG) anchored to the solid phase. In the case of APHRODITE, MBs will serve as the solid phase. This approach differs from the common practice of immobilizing antibodies on the microfluidic channel walls, offering several advantages:

- The reusability of the microfluidic network for multiple successive assays, as MBs can be temporarily captured within the channel during the assay and subsequently removed.
- Versatility, as different target analytes can be consecutively assayed in the same microfluidic network by selecting the appropriate reagents.
- Multiplexing allows for the simultaneous detection of cortisol and DHEA-S in a given oral fluid sample by trapping MBs coated with their respective specific antibody at distinct positions along the microfluidic channel.

To facilitate CL detection, a suitable CL cocktail containing luminol, hydrogen peroxide, and an enhancer was utilized. In the presence of HRP, which serves as a catalyst for the reaction, photon emission in the visible range is obtained.

### 2.2. APHRODITE System Design

**Figure 2:**
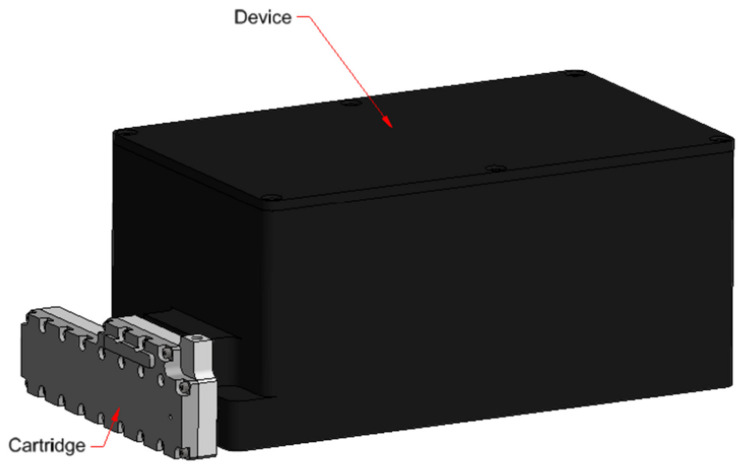
APHRODITE system CAD overview.

From its design phase, APHRODITE has been developed to meet the requirements for space applications and obtain the relevant certification for flight operations, such as compact dimensions, reduced weight, energy consumption, adherence to ISS safety requirements, and operation in microgravity and in the presence of relatively high levels of radiation [47]. The design, shown in Figure 2, makes it possible to minimize the weight and volume of waste material, as well as the cold stowage (6 ± 4 °C) space for storing the reagents until the time of analysis. APHRODITE’s main subsystems consist of:

- A disposable cartridge. It is a removable container with a series of reservoirs to host the reagents connected to fluidic subsystems. The cartridge also includes empty tanks for the introduction of the saliva sample to be analyzed and for the collection of waste solutions at the end of the analysis. The cartridge is 3D printed with a biocompatible material.
- A detection subsystem. It is the core of the system, and it is mainly composed of a glass microfluidic chip, which incorporates two channels made of a combination of medical-grade adhesive, glass substrate, and polymethyl methacrylate (PMMA). The microfluidic chip is comprised of two channels for redundancy; each channel accommodates a volume of 15 µL and exhibits a surface area measuring 245 mm^2^; the microfluidic connectors, which are at the interface between the inlet/outlet part of the chip and the microfluidic tubes from the fluidic dispensing sub-system, are specifically designed for the system and 3D printed.

An a-Si:H photosensor array composed of 30 thin-film photodiodes with a surface of 2 × 2 mm^2^ arranged in a 6 × 5 matrix was employed. The fabrication process involved several key steps to create the sensor array for the scientific application. Firstly, a transparent conductive layer was deposited using Magnetron Sputtering and subsequently patterned through wet etching to define the bottom electrode. Following this, the a-Si:H p-i-n stack, which constitutes the photodiode, was deposited using Plasma Enhanced Chemical Vapor Deposition. The optimization of the doping level, deposition time, and conditions, as well as the thickness of the a-Si:H layers, was carried out to achieve a low dark current level and high responsivity at the specific wavelength associated with the CL reaction. For the back electrode, a three-metal layer stack (consisting of 30 nm-thick Cr, 150 nm-thick Al, and another 30 nm-thick Cr layer) was deposited using magnetron sputtering. The sensor array was patterned using photolithography, involving both wet etching for the metal layers and reactive ion etching (RIE) for the a-Si:H layers. To provide insulation between the back metal and the front transparent conductive oxide (TCO) contacts, a 5 µm-thick SU-8 layer was spin-coated and patterned using UV lithography. An additional layer of TiW metal was deposited and patterned to define the external contact of the sensor array. Lastly, a 5 µm-thick SU-8 layer was deposited as a passivation layer. The a-Si:H photodiode technology demonstrates excellent optical detection performance, characterized by a low dark current, minimal noise, and high responsivity in the visible range [40,44]. Furthermore, these photodiodes exhibit remarkable radiation hardness, making them particularly suitable for in-orbit experiments. This sensor array was successfully tested in orbit during the AstroBio CubeSat mission [35].

**Figure 3:**
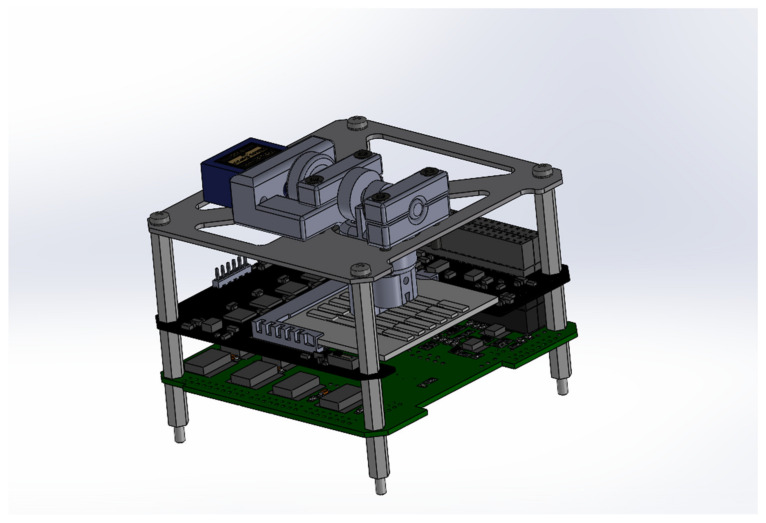
APHRODITE complete detection subsystem CAD.

A compact device for the generation of the magnetic field was developed, which is composed of a stepper motor that operates a cam able to control the position of two permanent magnets. The magnetic actuation subsystem, shown with its CAD in Figure 3, is designed to sequentially place two magnets within each channel. This sequential placement is essential as two different analytes are detected in different positions within the same channel. The magnets must be positioned and removed vertically. Importantly, the magnetic actuation subsystem is required to apply no discernible force on the microfluidic chip. The magnets need to be positioned directly on the chip’s surface. To fulfill this requirement, a cam and follower-based system has been developed. The cam and follower system comprises two distinct cams affixed to a shaft, which is in turn linked to a servo motor. For this application, a snail drop cam design has been chosen. This choice is based on the need for a substantial rise angle and dwell angle. The initial cam possesses a pronounced rise angle to facilitate a seamless transition to the high dwell angle. This design is crucial as the first magnet must remain in constant contact with the microfluidic chip, even during the transition phase.

**Figure 4:**
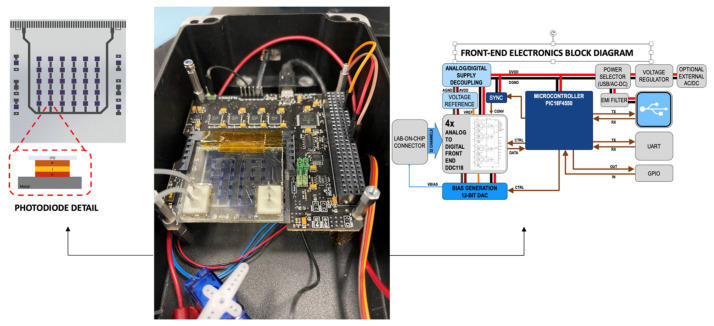
APHRODITE core detection subsystem setup and details (the magnetic subsystem is not present in this figure). (**Left**) photosensor array details. (**Center**) Core subsystems setup. (**Right**) Front-end electronic block diagram.

A low-noise front-end electronic board, shown in Figure 4, was employed for the acquisition and analogic-digital conversion of the photocurrent signals coming from the photosensors. The electronic board, named MARIE (Multichannel Array Readout Integrated Environment) was employed [28], which has been successfully tested in orbit during the AstroBio CubeSat mission [35]. The electronic board permits the acquisition of all the sensors of the on-chip array simultaneously, has 32 channels, a physical interface with the LoC Samtec MB1–150-S-02-SL card edge connector, a low-noise analog section with the circuit for the current readout, the bias voltage supply circuit (to operate the sensors, digitalize the signal and to time and control the analog part), and a digital section for the USB interface. MARIE’s digital part will also provide the digital control logic for the automatic or semi-automatic experiment execution, mediated by an auxiliary board that contains the analog drivers for the fluidic components (pumps and valve actuation, fluxmeter, and temperature sensor).

**Figure 5:**
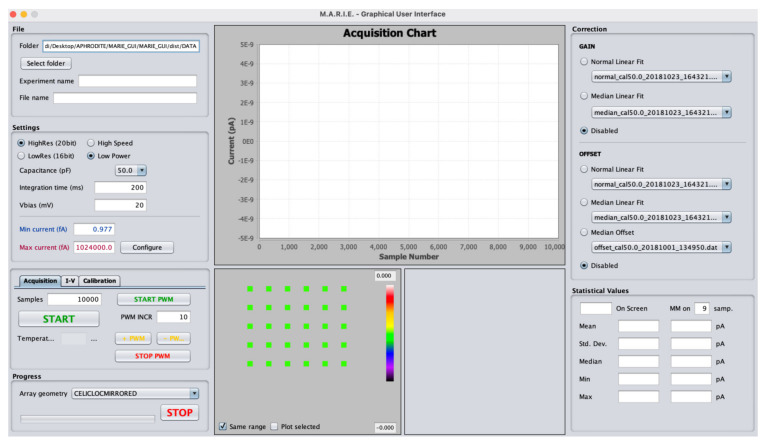
APHRODITE GUI.

A custom Java Graphic User Interface (GUI) has been set up to control all of the aspects of the system, including the calibration of the sensor array, the movement of the magnetic mechanism, the visualization, management, and storage of the acquired signal, and the automatic protocol execution. It is worth pointing out that the GUI is an integral part of the system as it shall allow for the complete control of the equipment, both in automatic and in a user-operated way, and it must satisfy the requirements for user interfaces aboard the ISS according to NASA reference documentation. The GUI has been developed in Java and runs under Windows, OS X, and Linux. A view of the GUI is reported in Figure 5 during the acquisition tests that will be later described. In particular, the GUI includes functions for setting the front-end acquisition parameters, setting the bias voltage, the definition of general acquisition parameters, the display of the acquired data, and data storage (both raw and pre-processed data). It also offers controls for system calibration and offset correction. Particular care has been devoted to data visualization as this function is of fundamental importance both in the testing and in the real operation phase. To this aim, a linear plot of all the acquired photocurrents (i.e., current vs. time) and a bi-dimensional intensity map have been implemented. The intensity map is necessary for the correct mapping of the acquired data on the correspondent reaction site on the lab-on-chip. In addition, real-time data statistics are elaborated and displayed. The histogram window displays the distribution of the current values measured by the selected photosensor, with no correction enabled. The letter and number that identifies the photosensor on the lab-on-chip is shown on the upper side of the window, with the number of the pin connected to it. The window refreshes every time a current value is acquired.

- A fluidic dispensing subsystem. The fluidic dispensing subsystem consists of tubing, fluidic connectors, miniaturized solenoid valves, miniaturized pumps, a flowmeter, and other elements necessary for the movement of fluids within the analytical device from the disposable cartridge to the detection subsystem.
- Control electronics and interface. The control and interface electronics are composed by an auxiliary board that deals with the management of the detection and fluidic subsystems for the automatic implementation of the protocol and with the connection to the onboard computer for the storage and transmission of the acquired data.
- Mechanical housing. Its primary functions are to contain the device ergonomically, to keep the detection subsystem in the dark, and to prevent the user from accidentally accessing the internal parts of the device; it includes the slot in which to insert the disposable cartridge and the USB interface.

### 2.3. APHRODITE Protocol

The correct execution of an immunological analysis involves a series of operations that must take place in sequence at specific time intervals. The protocol for recognizing and detecting the target analytes exploits a luminol/H_2_O_2_ reaction catalyzed by HRP, since it is a largely studied CL method and it can be easily implemented in miniaturized devices like LoC. The compactness of the final product’s volume and mass (1500 cm^3^, 1.5 kg) is a critical requirement to save the payload launch costs and operations and to use the system in future missions. Furthermore, the choice of such a reaction enabled us to have long-lasting CL signals, which is of the utmost importance for accurate dual-analyte detection.

The system is conceived to work on the ISS, and, because of it, the space radiation effect will not interfere in a determinant way with the measurements and system functions since a relatively low radiation dose, typically oscillating between 0.0002 and 0.0005 Gy per day on board the ISS [48], is expected. Nevertheless, APHRODITE is designed to be potentially used on a future Moon settlement, where the daily radiation dose is 2.6 times higher than on the ISS [49], or in a deep space trip to Mars. The importance of including in the system design components that are radiation-hard and that have already been validated in orbit on board ABCS [35] in the Van Allen Belts, a complex and dangerous environment in the context of radiation, is clear [50].

Another key point of the experiment relies on the data production and analytical method. Indeed, during the analysis, the CL emission is detected by an array composed of a few dozen solid-state photodetectors optically coupled to the microfluidic chip. Using discrete sensors instead of imaging devices (CCD or CMOS) has several advantages. First, it is possible to achieve higher signal-to-noise ratio levels and thus improve the LOD of the system. It is also possible to sample the CL signal many times per second as well as generate a limited amount of data, at least two orders of magnitude smaller than an imaging-based system that in any case would work at lower sampling rates. This is of the utmost importance for the effective use of such devices on the ISS and in future missions beyond LEO, when data management will be even more complex. The high frequency of sampling allows one to accurately monitor the kinetics of the CL signal, providing additional and complementary information to that obtained from the signal intensity alone [51].

**Figure 6:**
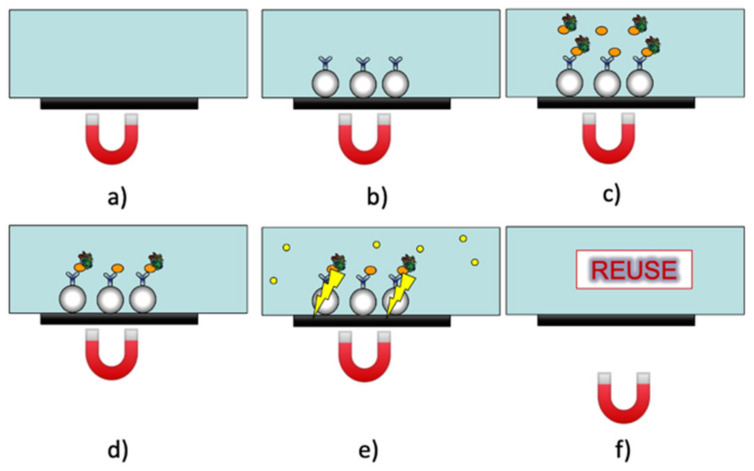
APHRODITE protocol representation for one analyte (e.g., cortisol). For the dual-analyte assay, the same procedure is simultaneously performed for the second analyte (e.g., DHEA-S) in a second position along the microfluidic channel. In (**a**) the magnet is positioned in the channel, then the MBs functionalized with a specific antibody (e.g., anti-cortisol) are inserted in (**b**); in (**c**), the labeled immunoreagent (e.g., cortisol-HRP) and the saliva sample interact with functionalized MBs and then the channel is washed in (**d**); the CL cocktail is injected in the channel and the CL signal is detected in (**e**); finally, the magnet is removed and the channel is washed for reuse in (**f**).

Regarding the protocol, schematized for a single measurement area section in Figure 6, the disposable cartridge contains the following reagents, necessary for carrying out two different analyses on the same sample:

- Antibody-functionalized MBs (specific for cortisol);
- Antibody-functionalized MBs (specific for DHEA-S);
- Labeled immunoreagent (specific for cortisol);
- Labeled immunoreagent (specific for DHEA-S);
- CL cocktail component A (luminol/enhancer);
- CL cocktail component B (peroxide);
- Accessory solution for washings during the assay procedure;
- Accessory solution for the final washing.

## 3. Materials and Methods

In this section, the materials and methods for the preliminary test on a dual analyte assay and the first integration test of the APHRODITE core subsystems are presented. Optimal experimental conditions, evaluated in preliminary experiments, were employed.

### 3.1. Chemicals

Polyclonal anti-cortisol and anti-DHEA-S antibodies produced in rabbits were purchased from Analytical Antibodies s.r.l. (Bologna, Italy), while HRP-labelled cortisol and DHEA-S used as tracers were bought from DiaMetra s.r.l. (Milano, Italy). The two-component (luminol/enhancer and oxidant) SuperSignal ELISA Femto CL substrate for HRP and the tosyl-activated superparamagnetic microbeads Dynabeads^®^ M-280 (diameter 2.8 µm) were obtained from Thermo Fisher Scientific, Inc. (Rockford, IL, USA). Cortisol, DHEA-S, HRP, and bovine serum albumin (BSA) were purchased from Sigma-Aldrich (St. Louis, MO, USA). All other chemicals were of analytical grade and were used as purchased. Solutions were prepared in phosphate-buffered saline (PBS) pH = 7.4.

### 3.2. Dual Analytes Assay Procedure

A preliminary evaluation of dual analyte assay feasibility was performed employing a simplified instrumental set-up, in which the detection subsystem was tested without the magnetic actuation system (i.e., the magnets were manually positioned on top of the interested channel with adhesive) and a syringe pump was used in place of the fluidic subsystem, allowing direct connection to the chip inlet. First, the channel was prepared for the immunoassay by loading the antibody-functionalized magnetic beads as follows. The second magnetic trap (i.e., the one that is farther with respect to the microfluidic channel inlet) was activated and 100 µL of a 0.2 mg mL^−1^ anti-cortisol MBs suspension in PBS was loaded in the channel at a 50 µL min^−1^ flow rate. After washing with 200 µL of PBS (50 µL min^−1^ flow rate), the first magnetic trap (i.e., the one that is closer to the microfluidic channel inlet) was also activated and 100 µL of a 0.2 mg mL^−1^ anti-DHEA-S MBs suspension in PBS were loaded in the channel at a 50 µL min^−1^ flow rate, followed by a second washing step with PBS (200 µL, 50 µL min^−1^ flow rate). The immunoreaction was started by injecting 100 µL of a standard solution at a 6.67 µL min^−1^ flow rate. Furthermore, 100 µL of a PBS solution containing cortisol-HRP (1:1000 (*v*/*v*) dilution) and DHEA-S-HRP (1:100 (*v*/*v*) dilution in PBS) was injected at a 6.67 µL min^−1^ flow rate, followed by 100 µL of PBS (16.67 µL min^−1^ flow rate) to move the immunoreagent in the channel to reach the MBs. After further washing with PBS (500 µL, 50 µL min^−1^ flow rate), CL detection was performed by injecting 200 µL of the HRP CL cocktail (the pre-mixed cocktail of the two components) at a 50 µL min^−1^ flow rate, acquiring the resulting CL signals during the injection of the cocktail. The CL signals were measured both in the correspondence of the two magnetic traps and in an intermediate position in the channel to assess the background signal due, e.g., to instrumental noise and spontaneous weak photon emission of the CL cocktail. Once the immunoassay was completed, the magnetic traps were deactivated and the MBs were removed by washing the channel with PBS (500 µL, 50 µL min^−1^ flow rate).

### 3.3. Integration Test Procedure

**Figure 7:**
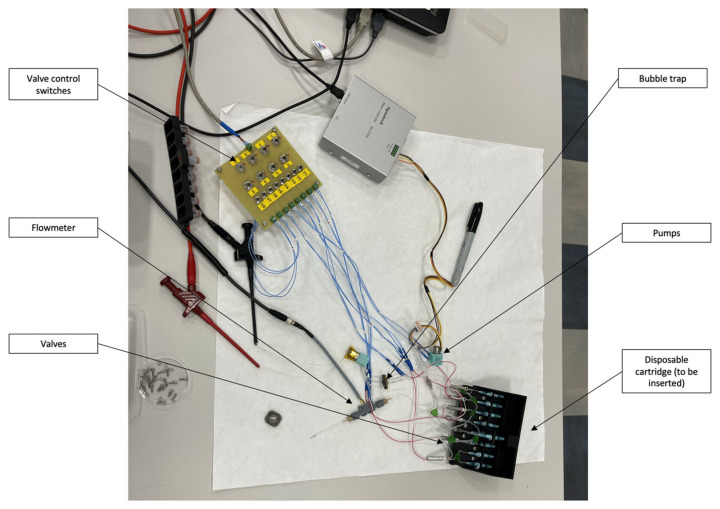
APHRODITE microfluidic setup for integration test.

**Figure 8:**
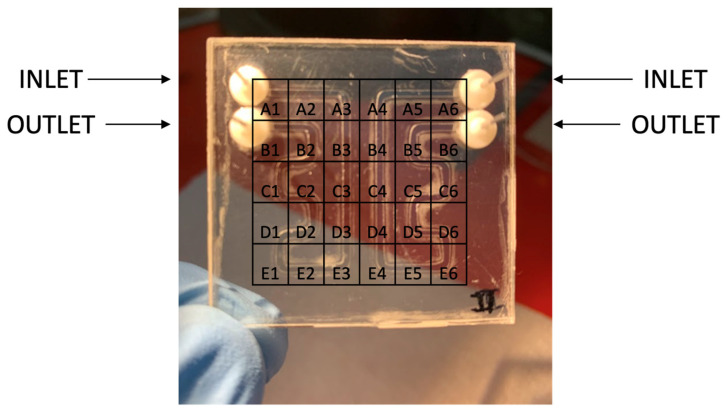
Microfluidic chip with pixel positioning and naming.

The presented results are based on an APHRODITE integration test measurement performed with a setup including the analytical subsystem, a fluidic dispensing subsystem with the channels, valves, flowmeter, bubble trap, and a first prototype of the disposable cartridge. The microfluidic setup used in the measurements is shown in Figure 7. The microfluidic part was directly connected to the core detection subsystem in a box containing the described electronics, microfluidic chip, and sensors (Figure 4). The chip setup is reported in Figure 8. The protocol for detecting one analyte (i.e., DHEA-S) was executed as follows. First, the system was primed by flowing 400 μL of PBS at a 60 μL min^−1^ flow rate. Then, upon activating the magnetic trap, 150 μL of a 20 mg mL^−1^ anti-DHEA-S MBs suspension in PBS was loaded at a 50 μL min^−1^ flow rate. Subsequently, upon washing with 200 μL of PBS (50 μL min^−1^ flow rate), 150 μL of DHEA-S-HRP (1:100 (*v*/*v*) diluted in PBS) was injected at a 6.67 µL min^−1^ flow rate, followed by 100 µL of PBS, (16.67 µL min^−1^ flow rate) in order to move the immunoreagent in the channel to reach the MBs, and then by 500 μL of PBS at 50 μL min^−1^, in order to wash away every residual from the channel properly. Subsequently, the CL cocktail mixture was generated into the fluidic system, by flowing 300 μL of component A and 300 μL of component B at 50 μL min^−1^ (mixing was obtained by acting on valves to alternate the reagent suction from the reservoir of each of the two components at 1 s intervals).

To get additional data regarding the minimum detectable CL, a partial system resetting with the insertion of 200 μL of MBs already reacted with the conjugate and 400 μL of extra substrate A and B (200 μL A + 200 μL B) from the cartridge testing the mixing protocol into the channel was accomplished.

## 4. Results

The data and results presented in this section of the paper have been acquired by exploiting two different configurations. The first is a simplified fluidic circuit; the experiment is here named dual analytes assay. This is an assay of reference. The second is the APHRODITE prototype (Figure 7), used to check the main subsystems’ functioning as a whole. Here, the experiment is named the APHRODITE integration test. All the tests were performed using samples at an analyte concentration of zero in order to check the response of the system in terms of maximum CL signal.

This first experiments with the APHRODITE core subsystems were made to check the validity of the concept and its initial performance.

### 4.1. Dual Analytes Assay Results

**Figure 9:**
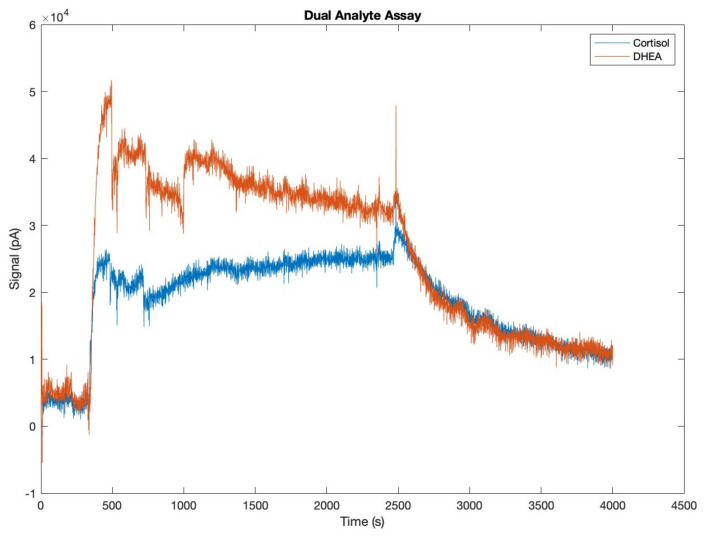
Dual analyte assay output signal (raw data).

A duplex competitive CL-immunoassay was developed for the simultaneous detection of cortisol and DHEA-S. The assays were based on the competition of DHEA-S and cortisol present in the sample with DHEA-S and cortisol conjugated to HRP for binding anti-DHEA-S and anti-cortisol antibodies, respectively, immobilized onto magnetic beads. By flowing the proper CL cocktail, it was possible to obtain CL signals that were monitored from the photosensors aligned in correspondence with the captured magnetic bead population (Figure 9). By spatially separating reaction areas corresponding to the detection of the two target analytes, it was possible to distinguish the signals relative to cortisol and DHEA-S. According to the competitive immunoassay principle, the light emission intensity from magnetic beads is expected to gradually decrease as the concentration of the respective analyte in the sample increases.

In the preliminary tests to evaluate the feasibility of the assay, a blank sample (zero analyte concentration) was analyzed, corresponding to the highest analytical signal obtained in the calibration curve (B_0_). Signals were acquired starting from the time of injection of the CL cocktail. In the initial phase, the CL signal was equal to the background. As soon as the cocktail reached the position where it came into contact with the HRP-analyte conjugate captured by the beads, the CL reaction started and a rapid increase in the signal was measured. The signal then remained almost stable until all the cocktail flowed inside the microchannel and began to decrease when the cocktail was finished. The test therefore demonstrated that it was possible to simultaneously detect the two target analytes in a single sample through a unique analysis. The difference in maximum signals achievable by the two target analytes was due to the different dilutions at which the analyte-HRP conjugates were used.

Indeed, the dilutions of the reagents used in the immunoassays had been previously optimized for obtaining high detectability within the range of the concentrations of physiological interest for the two analytes.

### 4.2. APHRODITE Integration Test Results

Following the design, fabrication, and testing of the APHRODITE subsystem’s primary components, a preliminary measurement session involving the integrated device’s key subsystems was conducted. This experiment included the execution of the protocol for DHEA-S detection without a saliva sample and demonstrated the successful operation of the core subsystems, encompassing microfluidic channels, photosensors, valves, and cartridge components.

**Figure 10:**
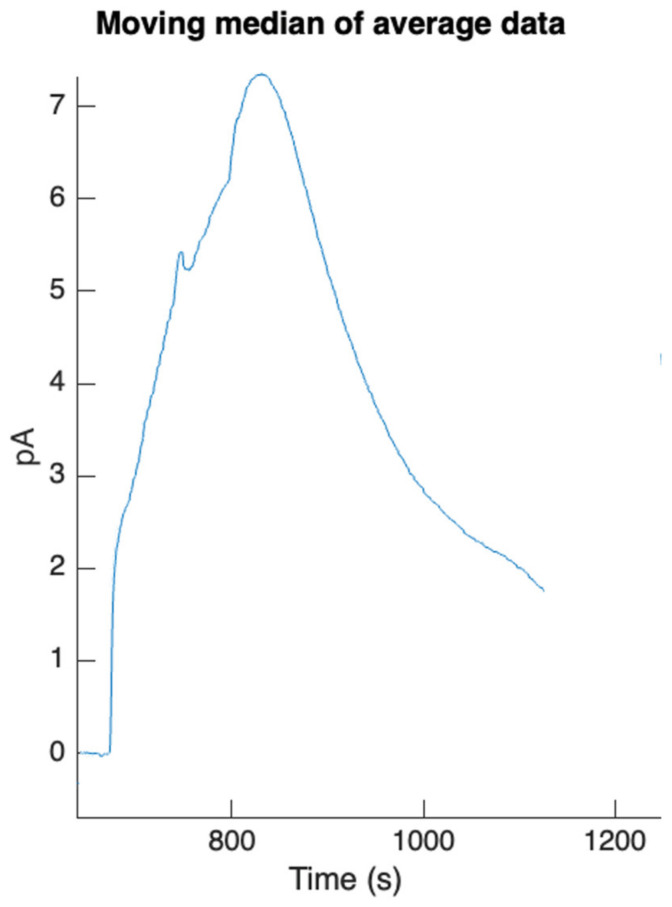
DHEA-S run output signal (moving median of average data) [37].

The system’s output data, displayed on the PC GUI, comprised a CL signal with a peak value of 7.5 pA. Figure 10 illustrates the moving median of the acquired data during the experiment’s initial run, highlighting the signal peak. To determine the signal peak precisely, the flow of the CL cocktail was halted at t = 869 s. The rationale behind this is that, under stop-flow conditions, the signal initially rises due to the extended residence time of the cocktail at the detection site, allowing for complete substrate utilization. During laminar flow, on the other hand, a portion of the cocktail may not reach the MBs due to the limited diffusion coefficient. Following the peak, the signal decays in a roughly exponential manner, as discussed in the subsequent paragraph.

**Figure 11:**
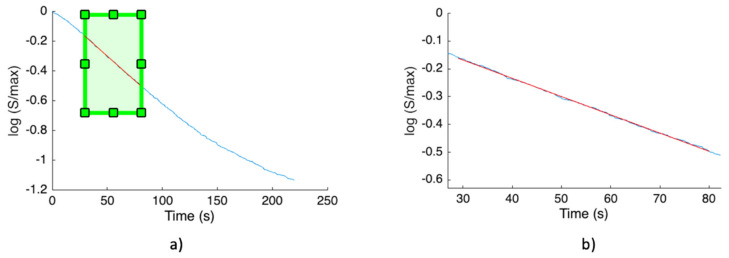
(**a**) Details of the output signal during stop flow condition. (**b**) The exponential decay of the output signal highlighted by the selected red part of the signal.

Figure 11 highlights the exponential decay of CL emission in the stop-flow condition mentioned above. The observed signal decrease aligns with the typical behavior observed in CL reactions under stop-flow conditions, and its evaluation is very important, as the CL decay time constant in stop-flow conditions correlates with the analyte concentration. With a high HRP concentration, as observed in the experiment, the reagents are consumed rapidly, resulting in a strong but short-lived signal. Conversely, a low HRP concentration produces a weak but slowly diminishing signal.

**Figure 12:**
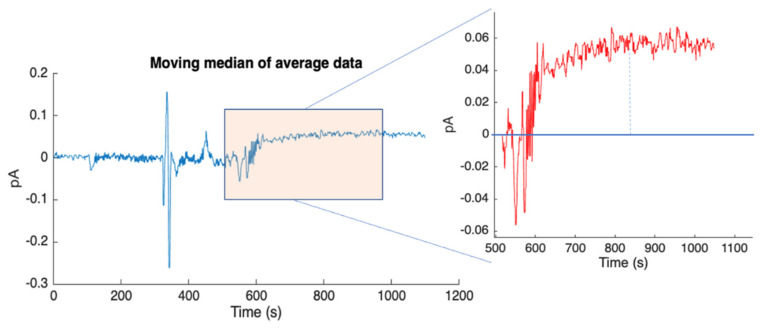
Moving the median of the acquired signal on pixel D4 for the minimum detectable CL signal.

To evaluate the detection system’s sensitivity, a test to determine the minimum CL signal was conducted. This test was performed without using functionalized MBs. Cocktail components A and B were introduced into the chip following the standard fluidic protocol, omitting the insertion of functionalized MBs into the channel. The absence of HRP to catalyze the CL reaction results in a minimum CL signal intensity due to a slow spontaneous reaction between cocktail components occurring even in the absence of the catalyzer. Consistent with the previous paragraph, the observed signal appears as an increase in the background, which remains relatively constant due to the extremely low rate of CL cocktail consumption under these conditions. It is noteworthy that, due to the competitive nature of the assay, this condition would correspond to the maximum concentration of the target analyte in the saliva sample. The results are shown in Figure 12, where a photocurrent signal of 0.07 pA is observed, corresponding to the minimum expected luminous intensity. The ability to detect this background signal with a favorable signal-to-noise ratio is crucial, as it indicates that the detection system, including the a-Si:H sensors and the front-end electronics, does not impose limitations on the system’s overall performance. These measurements provided the first insights into the system’s performance, establishing both the maximum and minimum CL signals detectable under the contrasting conditions of analyte absence or presence at high concentrations, respectively.

## 5. Conclusions

The APHRODITE design has been validated through some preliminary measurements and the integration of the core subsystems. The next steps will be to integrate a final ground model in order to make new measurements with saliva with known concentrations of cortisol and DHEA-S and to start the test campaign in order to qualify the device, a so-called flight model, to fly to the ISS.

Once validated in flight, APHRODITE could immediately be used in many studies conducted on the ISS to investigate the effect of the space environment on the human organism. It could also be adapted to the determination of other biomarkers of interest, simply by supplying new disposable cartridges with the necessary reagents. The first integration and tests of the subsystems show that the design concept works correctly with good performance levels.

## Data Availability

Data are contained within the article.

## Abbreviations

The following abbreviations are used in this manuscript:

a-Si:H	Hydrogenated amorphous silicon
ABCS	AstroBio CubeSat
AUX	Auxiliary
CL	Chemiluminescence
BSA	Bovine serum albumin
DHEA	Dehydroepiandrosterone
GUI	Graphic User Interface
ISS	International Space Station
LEO	Low Earth Orbit
LoC	Lab-on-Chip
LOD	Limit Of Detection
MARIE	Multichannel Array Readout Integrated Environment
MB	Microbeads
PBS	Phosphate-buffered saline
PCB	Printed circuit board
PMB	Paramagnetic microbeads
PMMA	Polymethyl methacrylate
RIE	Reactive Ion Etching
